# Oral Lactate Administration Additively Enhances Endurance Training-Induced Increase in Cytochrome C Oxidase Activity in Mouse Soleus Muscle

**DOI:** 10.3390/nu12030770

**Published:** 2020-03-14

**Authors:** Kenya Takahashi, Yu Kitaoka, Ken Yamamoto, Yutaka Matsunaga, Hideo Hatta

**Affiliations:** 1 Department of Sports Sciences, The University of Tokyo, 3-8-1 Komaba, Meguro-ku, Tokyo 153-8902, Japan; aynekihsahakat@gmail.com (K.T.); yamaken.kenken@icloud.com (K.Y.); y_matsunaga@idaten.c.u-tokyo.ac.jp (Y.M.); 2 Department of Human Sciences, Kanagawa University, 3-27-1 Rokkakubashi, Kanagawa-ku, Yokohama, Kanagawa 221-8686, Japan; kitaoka@kanagawa-u.ac.jp

**Keywords:** lactate, mitochondria, monocarboxylate transporter, skeletal muscle

## Abstract

We tested the hypothesis that oral lactate supplementation increases mitochondrial enzyme activity given the potential role of lactate for inducing mitochondrial biogenesis. In this study, mice were assigned to a saline-ingested sedentary group (S+S; *n* = 8), a lactate-ingested sedentary group (L+S; *n* = 9), a saline-ingested training group (S+T; *n* = 8), and a lactate-ingested training group (L+T; *n* = 8). Mice in the S+S and S+T groups received saline, whereas mice in the L+S and L+T groups received sodium lactate (equivalent to 5 g/kg of body weight) via oral gavage 5 days a week for 4 weeks. At 30 min after the ingestion, mice in the S+T and L+T groups performed endurance training (treadmill running, 20 m/min, 30 min, 5 days/week). At 30 min after lactate ingestion, the blood lactate level reached peak value (5.8 ± 0.4 mmol/L) in the L+S group. Immediately after the exercise, blood lactate level was significantly higher in the L+T group (9.3 ± 0.9 mmol/L) than in the S+T group (2.7 ± 0.3 mmol/L) (*p* < 0.01). Following a 4-week training period, a main effect of endurance training was observed in maximal citrate synthase (CS) (*p* < 0.01; S+T: 117 ± 3% relative to S+S, L+T: 110 ± 3%) and cytochrome c oxidase (COX) activities (*p* < 0.01; S+T: 126 ± 4%, L+T: 121 ± 4%) in the plantaris muscle. Similarly, there was a main effect of endurance training in maximal CS (*p* < 0.01; S+T: 105 ± 3%, L+T: 115 ± 2%) and COX activities (*p* < 0.01; S+T: 113 ± 3%, L+T: 122 ± 3%) in the soleus muscle. In addition, a main effect of oral lactate ingestion was found in maximal COX activity in the soleus (*p* < 0.05; L+S: 109 ± 3%, L+T: 122 ± 3%) and heart muscles (*p* < 0.05; L+S: 107 ± 3%, L+T: 107 ± 2.0%), but not in the plantaris muscle. Our results suggest that lactate supplementation may be beneficial for increasing mitochondrial enzyme activity in oxidative phenotype muscle.

## 1. Introduction

Mitochondria play a fundamental role in producing the cell fuel ATP. Given that increased mitochondrial content contributes to the improvement of exercise capacity [1,2] and prevention of numerous diseases [3,4,5], enhancing mitochondrial content is of great importance not only for athletic population, but also for ordinary people. Although exercise training is the most potent physiological inducer of mitochondrial biogenesis in skeletal muscle [6], lack of time has been reported as the leading barrier to regular exercise participation [7,8,9]. Hence, effective strategies to enhance mitochondrial biogenesis are required.

Lactate, once recognized as merely a product of glycogenolysis, is now seen as a carbohydrate fuel source for oxidative muscles [10,11,12]. From this point of view, the acute effects of lactate supplementation on exercise performance have been examined [13,14,15,16,17]. Interestingly, growing evidence suggests that lactate acts as a signaling molecule and contributes to mitochondrial adaptations in skeletal muscle [18,19]. We recently reported that daily lactate injection (1 g/kg BW of sodium lactate), which elevated blood lactate concentration to 12.7 ± 1.3 mmol/L, increased mitochondrial enzyme activity in mouse skeletal muscle [20]. This led us to hypothesize that oral lactate supplementation may enhance the exercise-induced mitochondrial adaptations in skeletal muscle and, therefore, would be an effective strategy for those who do not have time to participate in sufficient exercise.

In this study, we first examined the effects of 4-week oral lactate ingestion with or without endurance training on mitochondrial enzyme activities (CS and COX) and lactate transport proteins (monocarboxylate transporter (MCT) 1 and 4). Furthermore, we investigated the effects of oral lactate ingestion on acute phosphorylation responses of key kinases (AMPK, ACC, p38 MAPK, CaMKII), which are considered to be important in exercise-induced mitochondrial adaptations [21].

## 2. Materials and Methods 

### 2.1. Animals

Male ICR (Institute of Cancer Research) mice (8-week-old; Japan SLC, Inc., Tokyo, Japan) were used in this study. Animals were housed individually in a room, where temperature was maintained at 22 °C with a 12 h light and 12 h dark cycle (dark: 7:00 to 19:00). Standard laboratory chow (MF; Oriental Yeast, Tokyo, Japan) and water were provided ad libitum. All experiments were approved by the Animal Experimental Committee of The University of Tokyo (approval number: 27-14).

### 2.2. Experimental Design

#### 2.2.1. Four-Week Experiment

Animals were randomly allocated to one of four groups as follows: a saline-ingested sedentary group (S+S; *n* = 8), a lactate-ingested sedentary group (L+S; *n* = 9), a saline-ingested training group (S+T; *n* = 8), and a lactate-ingested training group (L+T; *n* = 8). Mice in the S+S and S+T groups received saline, whereas mice in the L+S and L+T groups received sodium lactate (equivalent to 5 g/kg of body weight) via oral gavage 5 days a week for 4 weeks. At 30 min after the ingestion, mice in the S+T and L+T groups performed a treadmill running at a speed of 20 m/min for 30 min. On the first day of ingestion, blood lactate concentration in the S+S, S+T, and L+T groups was measured using a portable analyzer (Lactate Pro 2, Arkray, Kyoto, Japan). At 24 hours after the last ingestion, mice were euthanized by blood removal from inferior vena cava under isoflurane inhalation. The plantaris, soleus, and heart muscles were taken, snap-frozen in liquid nitrogen, and stored at −80 °C.

#### 2.2.2. Single Bout Experiment

Animals were assigned as follows: a saline-ingested sedentary group (S+S; *n* = 8), a lactate-ingested sedentary group (L+S; *n* = 8), a saline-ingested exercise group (S+E; *n* = 8), and a lactate-ingested exercise group (L+E; *n* = 8). Mice in the S+S and S+E groups received saline, whereas mice in the L+S and L+E groups received sodium lactate (equivalent to 5 g/kg of body weight) via oral gavage. At 30 min after the ingestion, mice in the S+E and L+E groups performed a treadmill running at a speed of 20 m/min for 30 min. At 60 min after the ingestion (i.e. immediately after the endurance exercise for the S+E and L+E groups), mice were euthanized by blood removal from inferior vena cava under isoflurane inhalation. The plantaris and soleus muscles were quickly taken, snap-frozen in liquid nitrogen, and stored at −80 °C.

### 2.3. Analytical Methods

#### 2.3.1. Mitochondrial Enzyme Activity

Muscle specimens were homogenized using a μT-01 beads crasher (TITEC, Saitama, Japan) in 100 times (vol/wt) ice-cold homogenization buffer (100 mM monopotassium phosphate, pH 7.3). The sample homogenates were freeze-thawed twice using liquid nitrogen to disrupt the mitochondrial membrane. After centrifugation at 600 g for 10 min at 4 °C, the supernatant was collected and used for enzyme assay. The maximal activities of CS and COX were measured spectrophotometrically according to established protocols [22,23].

#### 2.3.2. Western Blotting

Whole plantaris and soleus muscles were homogenized in 30 times (vol/wt) radio immunoprecipitation assay (RIPA) buffer (50 mM Tris-HCl (pH 7.4), 150 mM NaCl, 0.25% deoxycholic acid, 1% NP-40, and 1 mM ethylenediaminetetraacetic acid (EDTA)) supplemented with protease inhibitor cocktail (cOmplete Mini, EDTA-free, Roche Applied Science, Mannheim, Germany) and phosphatase inhibitor cocktail (PhosSTOP, Roche Applied Science, Mannheim, Germany). The homogenates were rotated on ice for 60 min. After centrifugation at 1,500 g for 20 min at 4°C, the supernatant was collected. The protein concentration of each sample was quantified using the bicinchoninic acid (BCA) protein assay (TaKaRa BIO INC., Shiga, Japan). Equal amounts of protein (10 μg) were separated using standard sodium dodecyl sulfate-polyacrylamide gel electrophoresis (SDS-PAGE; 7.5–10%) procedure. Proteins were transferred to polyvinylidene difluoride (PVDF) membranes, and western blotting was carried out according to the protocol described in our previous study [20]. Antibodies used in the present study are listed in supplementary materials (Table S1). Blots were scanned using ChemiDoc XRS (Bio-Rad Laboratories, Hercules, CA, USA) and quantified using Quantity One (version 4.5.2, Bio-Rad). Consistent loading was verified by ponceau staining.

### 2.4. Statistical Analysis

All data are presented as means ± standard error of means (SEM). Welch’s *t*-test was used to examine the difference in blood lactate concentration immediately after the exercise. Two-way analysis of variance (ANOVA) was performed to examine the interaction and the main effects of lactate ingestion and endurance training in the 4-week experiment, or lactate ingestion and endurance exercise in the single bout experiment. Because there was no significant interaction between the two factors, post-hoc multiple comparison test was not performed. All statistical analyses were performed using GraphPad Prism (Ver. 7.0, Macintosh, GraphPad Software, La Jolla, CA, USA). Statistical significance was defined as *p* < 0.05. Details of F values and degrees of freedom are provided in supplementary materials (Table S2).

## 3. Results

### 3.1. Four-Week Experiment

#### 3.1.1. Blood Lactate Level after Lactate Ingestion

**Figure 1:**
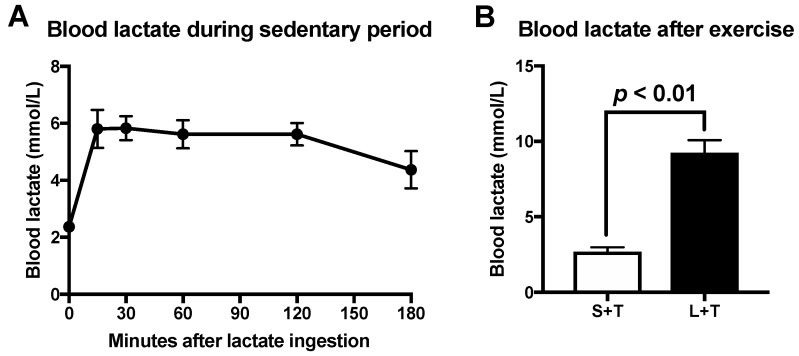
Blood lactate concentration following lactate ingestion during sedentary period (**A**). Blood lactate concentration immediately after the exercise (**B**). Data are presented as means ± SEM (*n* = 8).

On the first day of the ingestion, we confirmed blood lactate concentration. Peak blood lactate concentration of the L+S group reached 5.8 ± 0.4 mmol/L 30 min after lactate ingestion (Figure 1A). In addition, blood lactate level immediately after the exercise was significantly higher in the L+T group (9.3 ± 0.9 mmol/L) than in the S+T group (2.7 ± 0.3 mmol/L) (Figure 1B; *p* < 0.01).

#### 3.1.2. Body and Muscle Weights, and Energy Intake

Following the 4-week experimental period, body weight, plantaris muscle weight, soleus muscle weight and energy intake did not differ significantly (Table 1). 

#### 3.1.3. Mitochondrial Enzyme Activity Following 4-week Lactate Ingestion

**Figure 2:**
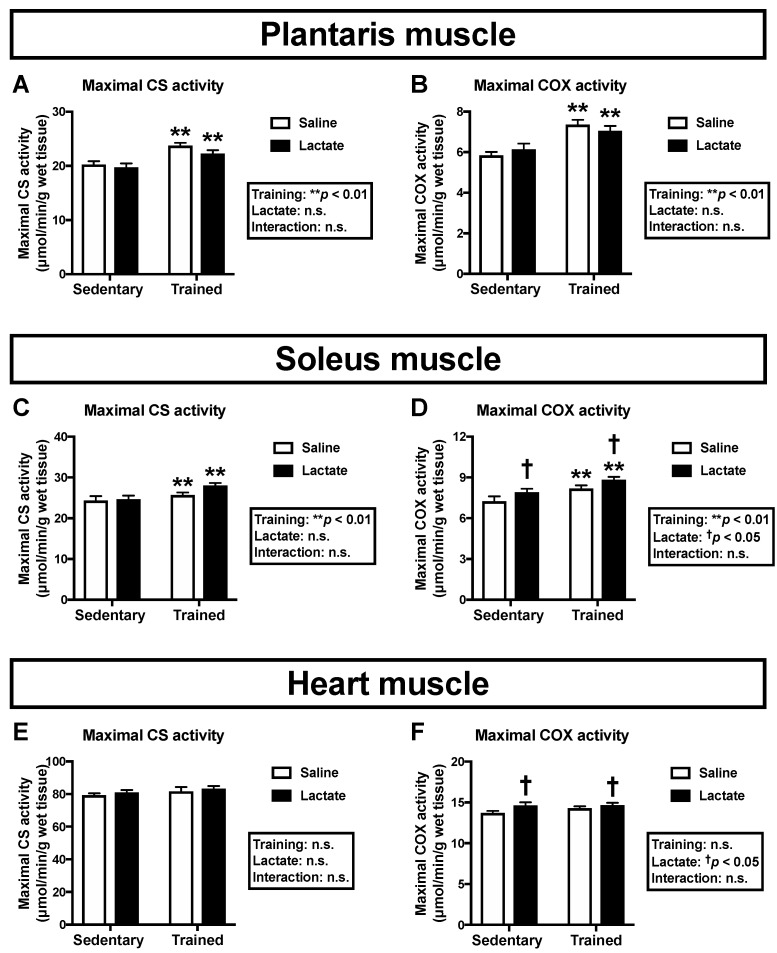
Maximal activities of CS (**A**, **C**, **E**) and COX (**B**, **D**, **F**) in the plantaris muscle (**A**, **B**), the soleus muscle (**C**, **D**), and the heart muscle (**E**, **F**) following 4-week lactate ingestion. Data are presented as means ± SEM (*n* = 8–9). ** *p* < 0.01: main effect of endurance training. ^†^ *p* < 0.05: main effect of lactate ingestion.

To clarify the effects of lactate ingestion on mitochondrial enzyme activity, we assessed maximal CS and COX activities. Endurance training had a positive main effect on maximal activities of CS and COX in both the plantaris muscle (*p* < 0.01; Figure 2A,B) and the soleus muscle (*p* < 0.01; Figure 2C,D). Additionally, lactate ingestion had a positive main effect on maximal COX activity in the soleus muscle (*p* < 0.05; Figure 2D), but not in the plantaris muscle (Figure 2B). Given that mouse soleus muscle comprises more oxidative phenotype fibers than the plantaris muscle, we further analyzed the heart muscle, which mostly comprises oxidative fibers and is a major consumer of lactate. Although exercise training had no effect on maximal activities of CS (Figure 2E) and COX (Figure 2F), lactate ingestion had a main effect (positive effect) on maximal COX activity in the heart muscle (*p* < 0.05; Figure 2F).

#### 3.1.4. MCT Protein Contents Following 4-Week Lactate Ingestion

**Figure 3:**
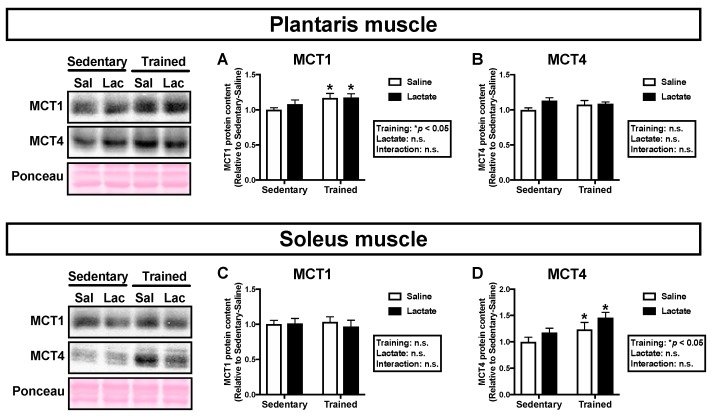
Protein contents of MCT1 (**A**, **C**) and MCT4 (**B**, **D**) in the plantaris muscle (**A**, **B**) and the soleus muscle (**C**, **D**) following 4-week lactate ingestion. Data are presented as means ± SEM (*n* = 8–9). * *p* < 0.05: main effect of endurance training.

Because the transport of lactate across the sarcolemmal membrane is facilitated by MCT1 and MCT4, we analyzed MCT protein contents in the skeletal muscle [24]. Endurance training had a positive main effect on MCT1 protein content in the plantaris muscle (*p* < 0.05; Figure 3A) and MCT4 protein content in the soleus muscle (*p* < 0.05; Figure 3D). Lactate ingestion had no significant effect on MCT protein contents in either the plantaris muscle (Figure 3A,B) or the soleus muscle (Figure 3C,D).

### 3.2. Single Bout Experiment

#### Phosphorylation State of Protein Associated with Mitochondrial Adaptations

**Figure 4:**
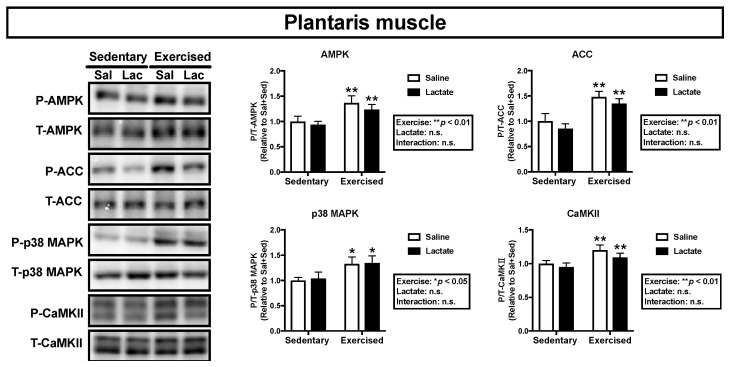
Phosphorylation of intramuscular signaling kinases in the plantaris 60 min after lactate ingestion (i.e., immediately after the exercise). Data are presented as means ± SEM (*n* = 8). * *p* < 0.05, ** *p* < 0.01: main effect of endurance exercise.

**Figure 5:**
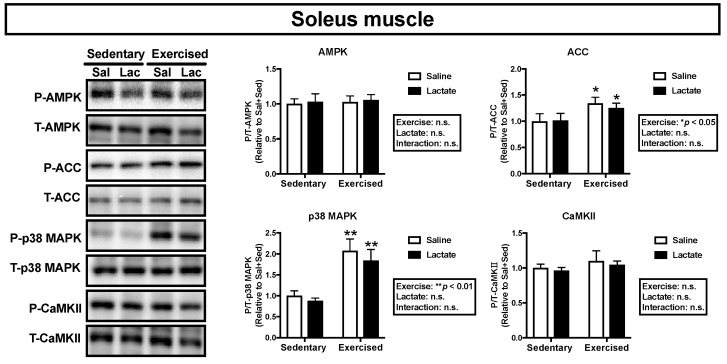
Phosphorylation of intramuscular signaling kinases in the soleus muscle 60 min after lactate ingestion (i.e., immediately after exercise). Data are presented as means ± SEM (*n* = 8). * *p* < 0.05, ** *p* < 0.01: main effect of endurance exercise.

We assessed phosphorylation of the key proteins, AMPK, ACC, p38 MAPK, and CaMKII, which are associated with mitochondrial adaptations [21]. Endurance exercise had a main effect on phosphorylation of ACC and p38 MAPK in the plantaris muscle (ACC: *p* < 0.01, p38 MAPK: *p* < 0.01; Figure 4) and the soleus muscle (ACC: *p* < 0.05, p38 MAPK: *p* < 0.01; Figure 5) and on phosphorylation of AMPK and CaMKII in the plantaris muscle (AMPK: *p* < 0.05, CaMKII: *p* < 0.01; Figure 4). However, lactate ingestion had no significant effect on phosphorylation of these proteins (Figure 4 and Figure 5).

## 4. Discussion

### 4.1. Mitochondrial Enzyme Activity Following Oral Lactate Administration

Although previous studies have suggested that lactate serves as a signaling molecule to induce physiological adaptations, little is known about the effects of oral lactate administration. The current study’s main finding is that oral lactate administration resulted in higher maximal COX activity in the soleus and heart muscles. Our results highlight the potential of oral lactate supplementation to enhance mitochondrial enzyme activity in oxidative phenotype muscle. Decreased mitochondrial enzyme activity in mouse skeletal muscle promoted fatigue development during electrical stimulation [25], while enhanced mitochondrial enzyme activity improved muscle resistance to fatigue [26]. Our observation that 4-week lactate ingestion resulted in enhanced COX activity in the soleus muscle may contribute to improved muscle function.

Oxidative phenotype muscle is a major site of lactate consumption and has larger protein content of MCT1, which mainly facilitates lactate uptake. In contrast, glycolytic muscle is a primal site of lactate production and possesses greater protein content of MCT4, which primarily facilitates lactate release from skeletal muscle [24,27,28]. Because these differences could affect mitochondrial adaptations, we assessed different phenotype muscles. In the present study, 4-week lactate ingestion enhanced mitochondrial enzyme activity in the soleus muscle, but not in the plantaris muscle. A previous study reported that incubation of L6 cells with lactate increased mRNA expression of peroxisome proliferator-activated receptor gamma coactivator 1-alpha (PGC-1α), which is a master regulator of mitochondrial adaptations [19]. In addition, we previously reported that a single intraperitoneal lactate injection increased PGC-1α mRNA expression in mouse gastrocnemius muscle [18]. More recently, we have shown a significant correlation between peak plasma lactate level during exercise and exercise-induced PGC-1α mRNA response in equine gluteus medius muscle [29], which consists of approximately 90% fast fibers [30], suggesting a lactate concentration-dependent increase in PCG-1α mRNA expression at least in glycolytic fibers. Mouse plantaris and gastrocnemius muscles consist of more than 90% fast fibers [31,32]. In our previous study, 3-week lactate injection, which elevated blood lactate concentration to 12.7 ± 1.3 mmol/L, enhanced mitochondrial enzyme activity in the plantaris muscle [20], whereas 4-week lactate ingestion, which increased blood lactate level to 5.8 ± 0.4 mmol/L, did not change mitochondria enzyme activity in the plantaris muscle in the present study. Collectively, a larger increase in blood lactate concentration may be required to enhance mitochondrial enzyme activity in the plantaris muscle. Contrary to the plantaris muscle, 4-week lactate ingestion enhanced maximal COX activity in the soleus muscle. In our previous study, maximal COX activity in the soleus muscle tended to be higher after 3-week lactate injection (*p* = 0.057) [20]. In human study, PGC-1α mRNA level in vastus lateralis muscle was increased at exercise intensities above the lactate threshold (LT), but not below the LT [33]. LT is an intensity, generally ranging from 2 to 4 mmol/L, where blood lactate level steeply increases [34]. Human skeletal muscles, such as vastus lateralis and gastrocnemius muscles, are composed of 40–50% slow fibers [35,36], which is similar to mouse soleus muscle (approximately 40% slow fibers) [31,32]. Taken together, blood lactate concentration to an upper physiological level might not be required to induce mitochondrial adaptations in mouse soleus muscle.

In the current study, the phosphorylation states of AMPK, ACC, p38 MAPK, and CaMKII—key kinases for mitochondrial adaptation [21]—did not change after lactate ingestion. This is in accordance with previous studies reporting no significant difference in those kinases after manipulating circulating lactate level [20,37,38], which suggests that those kinases are not associated with lactate-induced increases in mitochondrial enzyme activity. The precise mechanisms underlying enhanced mitochondrial enzyme activity after lactate administration remain unclear. Previous studies reported that lactate activates other signaling pathways by binding a lactate selective receptor, G-protein-coupled receptor (GPR81) [39,40]. Moreover, lactate was reported to promote secretion of TGF-β2 [41] and FGF21 [42] from adipose tissue and to increase blood BDNF level [43]. We assume that inter-organ communication caused by lactate contribute, in part, to enhanced mitochondrial enzyme activity.

Previous studies reported that CS and COX activities were highly correlated with mitochondrial content in human skeletal muscle [44], and that mitochondrial content was significantly correlated with exercise performance [2]. However, although it is generally considered that mitochondrial content and respiration increase in parallel [45], this is not the case in some studies [46,47]. Previous studies reported that training-induced increases in mitochondrial respiration did not parallel with changes in mitochondrial enzyme activities [48,49]. In another study, increases in mitochondrial content were not followed by an increase in mitochondrial respiration [50]. In addition, mitochondria change not only their content and respiratory capacity, but also their morphology in relation to the expression of dynamics regulatory proteins [51,52]. Therefore, effects of lactate on mitochondrial respiratory capacity and their morphology, as well as exercise performance should be clarified in the future studies.

### 4.2. Oral Lactate Administration and MCT Protein Contents

Previous studies have shown that high-intensity exercise training, which elevates circulating lactate, increased both MCT1 and MCT4 protein contents [53,54,55], which suggests increased circulating lactate enhances MCT protein contents. In the present study, lactate ingestion did not increase MCT protein contents. Similarly, we previously reported no difference in MCT protein contents after 3 weeks of daily lactate injection [20]. Our observations suggest that a mere elevation of circulating lactate does not increase MCT protein contents in skeletal muscle. 

Unlike our current observations, we previously reported that 3 weeks of post-exercise oral lactate administration increased MCT1 protein content and glycogen concentration in mouse skeletal muscle compared with exercise training alone [56], which suggests that lactate induces different adaptations depending on the timing of administration. Lactate oxidation is increased during exercise [57], whereas lactate partially serves as a substrate for glycogen formation after exercise [58]. Altogether, the differences in lactate metabolism during and after exercise, but not the degree of blood lactate concentration, may affect physiological adaptations.

We and others previously reported that AMPK activation by 5-aminoimidazole-4-carboxamide-1-beta-d-ribofuranoside (AICAR) injection increased MCT protein contents in rodent skeletal muscle [59,60]. In the current study, acute endurance exercise, but not lactate ingestion, increased phosphorylation of AMPK and ACC, indicators of AMPK activity. Additionally, 4-week endurance training enhanced MCT1 protein content in the plantaris muscle and MCT4 protein content in the soleus muscle. Although MCT protein contents are regulated by several mechanisms [61], AMPK activation is likely to be important for increasing MCT protein contents.

### 4.3. Effects of Metabolic Alkalosis

Lactate ingestion induces metabolic alkalosis [13,62] and metabolic alkalosis, induced by sodium bicarbonate ingestion, augments high-intensity exercise-induced increase in mitochondrial respiratory capacity in rat soleus muscle [63]. These observations imply that lactate ingestion induces mitochondrial adaptation via metabolic alkalosis. Additionally, sodium bicarbonate supplementation enhances high-intensity exercise-induced increases in blood lactate concentration and PGC-1α mRNA expression in humans [37]. Therefore, we assume that metabolic alkalosis-induced mitochondrial adaptation results from greater increase in blood lactate concentration.

### 4.4. Ingestion Volume and Future Perspective

Finally, in the current study, mice ingested 5 g/kg BW of lactate. A previous study reported no alteration in mitochondrial enzyme activity in rat skeletal muscle following ingestion of lactate (1 g/kg BW) with caffeine every other day for 5 weeks [64], which suggests that a certain amount of lactate is needed to enhance mitochondrial enzyme activity. Although lactate supplementation, with a view to providing fuel source, has been applied to humans [13,14,15,16,17], gastrointestinal distress was reported after consuming a large volume of lactate [14]. Therefore, future studies should determine the optimal doses of lactate and the efficacy of lactate in humans.

## 5. Conclusions

In the current study, lactate ingestion resulted in enhanced maximal COX activity in the soleus and the heart muscles, but not in the plantaris muscle. Our results suggest that lactate supplementation may be beneficial for increasing mitochondrial enzyme activity in oxidative phenotype muscle. Since our findings are limited to mitochondrial enzyme activity, effects of lactate on mitochondrial respiration and morphology, as well as exercise performance should be clarified in the future studies.

## Figures and Tables

**Table 1:** Initial and final body weights, soleus muscle weight, plantaris muscle weight, and energy intake in 4-week ingestion experiment.

	S+S (*n* = 8)	L+S (*n* = 9)	S+T (*n* = 8)	L+T (*n* = 8)
Initial body weight (g)	36.7 ± 0.5	36.4 ± 0.6	36.8 ± 0.4	36.4 ± 0.9
Final body weight (g)	40.6 ± 0.8	40.5 ± 0.5	41.2 ± 0.5	40.6 ± 0.7
Plantaris muscle (mg)	40.0 ± 1.3	41.7 ± 1.4	40.3 ± 1.6	41.3 ± 1.5
Soleus muscle (mg)	17.6 ± 0.6	16.8 ± 1.1	19.0 ± 0.8	18.6 ± 0.9
Energy intake (kcal/day)	18.2 ± 0.5	17.9 ± 0.5	18.8 ± 0.3	18.0 ± 0.5

Data are presented as means ± SEM.

## Supplementary Materials

The followings are available online at https://www.mdpi.com/2072-6643/12/3/770/s1, Table S1: Antibodies, Table S2: F values and degrees of freedom.
