# Supplementary material for: Oral Lactate Administration Additively Enhances Endurance Training-Induced Increase in Cytochrome C Oxidase Activity in Mouse Soleus Muscle
Source: Nutrients. 2020 Mar 14;12(3):770. doi: 10.3390/nu12030770 (PMC7146285; doi:10.3390/nu12030770)
Supplement: Supplementary file 1 [file nutrients-12-00770-s001.pdf]

Table S1: Antibodies

Primary antibody	Reference number; Manufacturer
MCT1	custom-made; Qiagen Japan, Tokyo, Japan
MCT4	custom-made; Qiagen Japan, Tokyo, Japan
Phosphorylated AMPK	no. 2513; CST Japan, Tokyo, Japan
Total AMPK	no. 2532; CST Japan, Tokyo, Japan
Phosphorylated ACC	no. 3661; CST Japan, Tokyo, Japan
Total ACC	no. 3662; CST Japan, Tokyo, Japan
Phosphorylated p38 MAPK	no. 9211; CST Japan, Tokyo, Japan
Total p38 MAPK	no. 9212; CST Japan, Tokyo, Japan
Phosphorylated CaMKII	no. 3361; CST Japan, Tokyo, Japan
Total CamKII	no. 611292; BD Biosciences Japan, Tokyo, Japan

Secondary antibody	Reference number; Manufacturer
Rabbit anti-goat IgG (H&L)	A106PU; American Qualex, San Clemente, CA, USA
Mouse anti-goat IgG (H&L)	A102PT; American Qualex, San Clemente, CA, USA

Table S2: F values and degrees of freedom

Blood lactate concentration after the exercise

F (DFn, DFd)	F (7, 7) = 8.372
P value	$p < 0.01$

Four-week experiment

Measurement items	Interaction F (DFn, DFd) *p* value	Training F (DFn, DFd) *p* value	Lactate F (DFn, DFd) *p* value
Initial body weight	F (1, 29) = 0.009506 not significant	F (1, 29) = 0.003213 not significant	F (1, 29) = 0.3086 not significant
Final body weight	F (1, 29) = 0.1232 not significant	F (1, 29) = 0.3011 not significant	F (1, 29) = 0.2795 not significant
Plantaris muscle	F (1, 29) = 0.003893 not significant	F (1, 29) = 0.007735 not significant	F (1, 29) = 1.223 not significant
Soleus muscle	F (1, 29) = 0.08501 not significant	F (1, 29) = 3.358 not significant	F (1, 29) = 0.4881 not significant
Energy intake	F (1, 29) = 0.27 not significant	F (1, 29) = 0.6512 not significant	F (1, 29) = 1.343 not significant

Plantaris muscle

Measurement items	Interaction F (DFn, DFd) *p* value	Training F (DFn, DFd) *p* value	Lactate F (DFn, DFd) *p* value
CS activity	F (1, 29) = 0.5378 not significant	F (1, 29) = 23.41 $p < 0.01$	F (1, 29) = 2.547 not significant
COX activity	F (1, 29) = 1.582 not significant	F (1, 29) = 25.28 $p < 0.01$	F (1, 29) = 0.0005285 not significant
MCT1	F (1, 29) = 0.4902 not significant	F (1, 29) = 5.813 $p < 0.05$	F (1, 29) = 0.6752 not significant
MCT4	F (1, 29) = 2.156 not significant	F (1, 29) = 0.1472 not significant	F (1, 29) = 3.213 not significant

Soleus muscle

Measurement items	Interaction F (DFn, DFd) *p* value	Training F (DFn, DFd) *p* value	Lactate F (DFn, DFd) *p* value
CS activity	F (1, 29) = 1.65 not significant	F (1, 29) = 8.772 $p < 0.01$	F (1, 29) = 2.744 not significant
COX activity	F (1, 29) = 0.004481 not significant	F (1, 29) = 11.97 $p < 0.01$	F (1, 29) = 6.222 $p < 0.05$
MCT1	F (1, 29) = 0.2577 not significant	F (1, 29) = 0.007857 not significant	F (1, 29) = 0.1283 not significant
MCT4	F (1, 29) = 0.05859 not significant	F (1, 29) = 6.437 $p < 0.05$	F (1, 29) = 3.902 not significant

Heart muscle

Measurement items	Interaction F (DFn, DFd) *p* value	Training F (DFn, DFd) *p* value	Lactate F (DFn, DFd) *p* value
CS activity	F (1, 29) = 0.005462 not significant	F (1, 29) = 1.708 not significant	F (1, 29) = 0.9463 not significant
COX activity	F (1, 29) = 1.028 not significant	F (1, 29) = 1.162 not significant	F (1, 29) = 5.149 $p < 0.05$

Single bout experiment

Plantaris muscle

Measurement items	Interaction F (DFn, DFd) *p* value	Training F (DFn, DFd) *p* value	Lactate F (DFn, DFd) *p* value
P/T-AMPK	F (1, 28) = 0.1001 not significant	F (1, 28) = 10.11 $p < 0.01$	F (1, 28) = 0.8613 not significant
P/T-ACC	F (1, 28) = 0.004061 not significant	F (1, 28) = 17.26 $p < 0.01$	F (1, 28) = 1.411 not significant
P/T-p38 MAPK	F (1, 28) = 0.005762 not significant	F (1, 28) = 7.08 $p < 0.05$	F (1, 28) = 0.06139 not significant
P/T-CaMKII	F (1, 28) = 0.2398 not significant	F (1, 28) = 7.786 $p < 0.01$	F (1, 28) = 1.609 not significant

Soleus muscle

Measurement items	Interaction F (DFn, DFd) *p* value	Training F (DFn, DFd) *p* value	Lactate F (DFn, DFd) *p* value
P/T-AMPK	F (1, 28) = 3.877e-005 not significant	F (1, 28) = 0.108 not significant	F (1, 28) = 0.1129 not significant
P/T-ACC	F (1, 28) = 0.1837 not significant	F (1, 28) = 5.677 $p < 0.05$	F (1, 28) = 0.09205 not significant
P/T-p38 MAPK	F (1, 28) = 0.0823 not significant	F (1, 28) = 25.25 $p < 0.01$	F (1, 28) = 0.7724 not significant
P/T-CaMKII	F (1, 28) = 0.009092 not significant	F (1, 28) = 1.17 not significant	F (1, 28) = 0.2606 not significant
